# Feasibility and Usability of Patch-based Continuous Cardiac Rhythm Monitoring in Comparison with Traditional Telemetry in Noncritically Ill Hospitalized Patients

**DOI:** 10.19102/icrm.2019.100901

**Published:** 2019-09-15

**Authors:** Ram Amuthan, Alicia Burkle, Steven Mould, John Tote, Molly Loy, Desiree Kirkwood, Josalyn Meyer, Shannon Pengel, Aaron C. Hamilton, Daniel J. Cantillon

**Affiliations:** ^1^Department of Internal Medicine, Cleveland Clinic Foundation, Cleveland, OH, USA; ^2^Department of Nursing, Cleveland Clinic Foundation, Cleveland, OH, USA; ^3^Electrophysiology Section, Department of Cardiovascular Medicine, Cleveland Clinic Foundation, Cleveland, OH, USA

**Keywords:** Alarm fatigue, cardiac telemetry, continuous cardiac monitoring, patch monitoring

## Abstract

Research on traditional cardiac telemetry demonstrates that excessive alarms are related to lead failures and noise-related interruptions. Patch-based continuous cardiac rhythm monitoring (CCRM) has emerged in outpatient ambulatory monitoring situations as a means to improve recording fidelity. In this study, patients hospitalized but not in the intensive care unit were simultaneously monitored via telemetry in parallel with the use of the Vital Signs Patch™ (VSP) CCRM system (LifeWatch Services, Rosemont, IL, USA), applying standardized monitoring and notifications provided by an off-site central monitoring unit (CMU). Among 11 patients (55% male; age: 66.8 ± 12.5 years), there were 42 CMU detections and 98 VSP detections. The VSP device was successfully applied by nursing with connectivity established in all 11 patients (100%). There were no VSP device–related adverse events or skin eruptions during the study. The CMU agreed with 59 (60%) of 98 VSP detections. Among those detections marked by disagreement 30 (77%) of 39 VSP detections were related to clinically meaningful arrhythmias (atrial: n = 9; ventricular: n = 7; brady-: n = 14) undetected by VSP due to noise. In two patients (18%), there were four clinically meaningful atrial fibrillation detections not recorded by the CMU. In conclusion, patch-based CCRM requires further development and review to replace traditional cardiac telemetry monitoring but could evolve into an appropriate method to detect clinically meaningful events missed by traditional methods if noise issues can be mitigated.

## Introduction

Several hospital-based studies to date have demonstrated that routine electrocardiographic telemetry monitoring results in a high volume of alarms without immediate clinical relevance.^1–3^ This has led to a form of clinical desensitization, referred to as alarm fatigue, which has been attributed to several negative clinical outcomes including death.^3^ At the Cleveland Clinic, the utilization of an off-site central monitoring unit (CMU) that applies standardized cardiac telemetry indications in hospitalized patients not admitted to the intensive care unit (ICU) was associated with improved clinical outcomes.^4^ The CMU provided accurate and early notification in 79% of emergency response team (ERT) activations, with a 93% return of spontaneous circulation when CMU monitoring technicians applied discretionary direct ERT communication in advance of an impending cardiopulmonary arrest.^4^ However, around 42% of CMU notifications were the result of occurrences such as lead failure and telemetry disruption.^4^ Such telemetry disruptions render patients vulnerable to undetected clinically relevant arrhythmias or hemodynamic events. The fidelity of electrode connections has been identified as a major contributor to telemetry disruptions in previous studies.^3–5^ Patch-based continuous cardiac rhythm monitoring (CCRM) has emerged in the outpatient setting as a useful clinical tool for the quantification and surveillance of bradyarrhythmias and tachyarrhythmias.^6,7^ These patch-based CCRM devices have been studied in the outpatient setting as an alternative to traditional Holter monitoring and have emerged as feasible and patient-friendly options for the detection of clinically meaningful arrhythmias.^8,9^ The present study sought to evaluate the feasibility and usability of the Vital Signs Patch™ (VSP) CCRM system (LifeWatch Services, Rosemont, IL, USA) in non-ICU–hospitalized patients against the outcomes achieved with the current standard of conventional telemetry monitoring. The primary goal of this research was to elucidate early feasibility and usability characteristics of the aforementioned VSP device (including any skin-related adverse events) and to investigate its capacity to detect clinically meaningful arrhythmias while potentially decreasing the number of alarms due to telemetry interruptions by way of its novel skin fixation mechanism.

## Materials and methods

### Patients

Between August 2015 and December 2015, 11 non-ICU–hospitalized patients underwent CCRM simultaneously with use of the VSP device and traditional telemetry systems in parallel, applying standardized monitoring criteria and notifications provided by a monitoring technician located at an off-site CMU to bedside nursing personnel using a previously published protocol.^4^ The CMU is referred to as “secondary” monitoring because it does not replace “primary” monitoring alarms in the form of audible alerts occurring at the nursing station, but rather aims to ensure that clinically important alarms are not missed by bedside nursing personnel. Pediatric patients, pregnant individuals, patients with internal or external defibrillators, patients with extensive skin damage or fresh surgical incisions on the chest, and patients in critical care areas were excluded from participation in this study. The present investigation followed the relevant institutional review board policies, and written informed consent was obtained from all patients. The initial single-center study plan called for a minimum of 10 patients receiving telemetry monitoring for the early feasibility and usability assessment, followed by a larger enrollment of 25 patients; however, this latter initiative was discontinued when the manufacturer discontinued the product as a result of evolving business considerations.

### Devices and monitoring protocols

**Figure 1:**
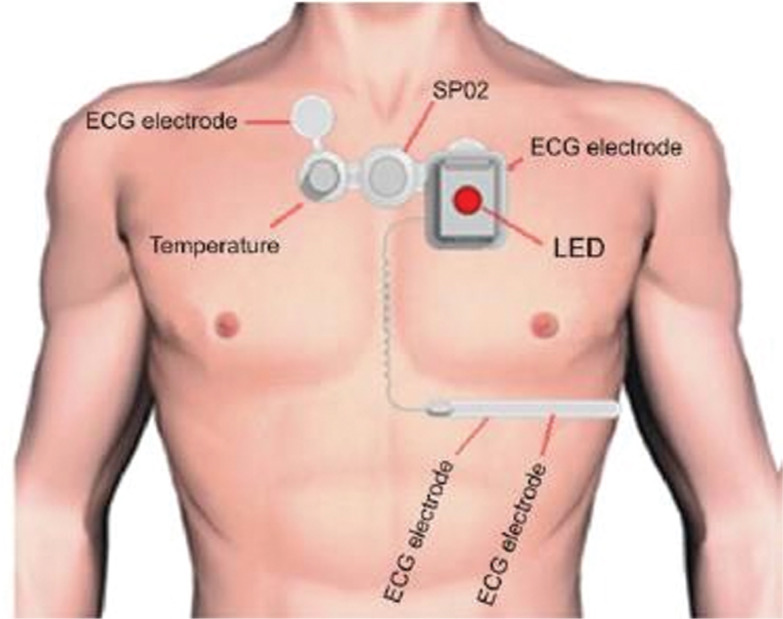
VSP monitor and components.

The VSP device considered in this study was a United States Food and Drug Administration–approved multichannel [three-lead electrocardiogram (ECG)] patch-based CCRM system. It is also designed to monitor and record blood saturation, body temperature, respiration, and blood pressure, but these parameters were not assessed in the present research **(Figure 1)**. The data from the VSP CCRM system were stored on the manufacturer’s secure server on the premises of the Cleveland Clinic and under its firewall in compliance with institutional data security policies. After the end of the study, the raw data underwent technical review for report generation and were made available to the investigators for review. The report included the specifics of arrhythmia detection and raw ECG waveforms for every instance of detection by the VSP CCRM system. The investigators compared data from the CMU flow sheet, which contained the specifics of conventional telemetry detections and included raw ECG waveforms of every detection.

### Analysis

The data from the VSP CCRM and the CMU using traditional telemetry were first aligned according to their timestamps and comparisons triggered by events detected on either platform were performed. For each triggered event, the rhythm strip was reviewed on both the triggered platform and the corresponding platform at the precise timestamp. The real-time CMU interpretation assigned by a monitoring technician utilized for clinical care was captured in the electronic medical record. An unblinded internal medicine resident physician acting as an investigator provided initial rhythm interpretation reads of the VSP detections as well as over-reads of the CMU detections, applying institutional standards.^4^ Each triggered event was either assigned a rhythm interpretation or designated as uninterpretable in the event of unacceptable noise or as a loss of ECG signal. A board-certified cardiac electrophysiologist was then provided blinded rhythm strips for final approval. Adjudicated interpretations of the VSP data and CMU interpretations were subsequently evaluated for agreement.

## Results

### Feasibility and usability

**Figure 2:**
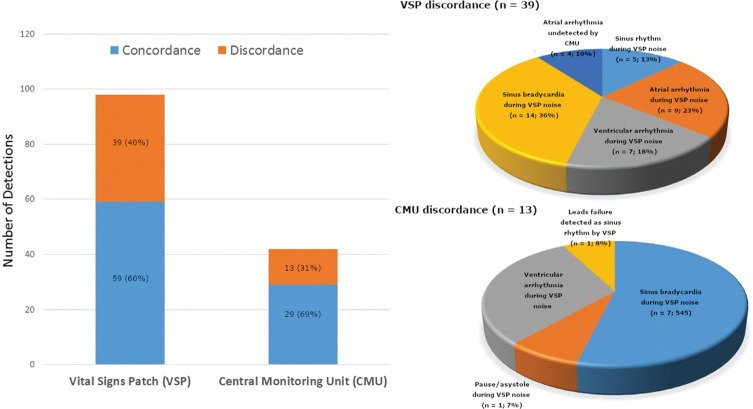
Total number of detections on the VSP (n = 98) and the CMU (n = 59), with respective distributions of concordance (blue) and discordance (orange) when compared with one another.

Eleven patients were monitored simultaneously via the VSP CCRM system and conventional cardiac telemetry monitoring using notifications provided by the CMU. **Table 1** shows the baseline clinical characteristics of the study cohort. The VSP device was successfully applied by nursing staff, with connectivity established in all 11 patients (100%). There were no VSP-related adverse events or skin eruptions that occurred during the period of the study. There were 42 triggered CMU detections and 98 triggered VSP detections **(Figure 2 and Table 2)**. In all 42 CMU detections, the real-time interpretation assigned by the monitoring technician agreed with the over-read interpretation assigned by the resident physician investigator as well as the blinded electrophysiologist over-read.

### VSP detections

**Figure 3:**
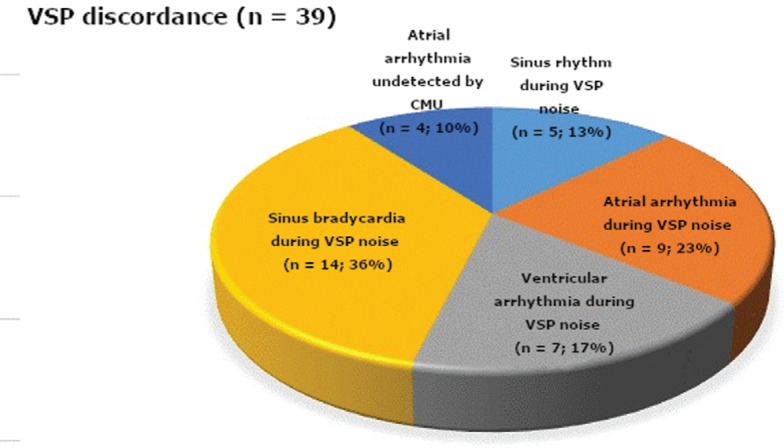
Pie graph characterizing the VSP detections that were discordant with the CMU (n = 39; 40%). The majority resulted from VSP noise detection during CMU detection of sinus rhythm (13%), atrial arrhythmia (23%), ventricular arrhythmia (18%), and sinus bradycardia (36%). However, four VSP detections (10%) resulted from atrial arrhythmias undetected by the CMU.

Out of the 98 VSP detections, agreements were established between the VSP and the CMU in 59 (60%) of the detections. The CMU did not agree with 39 (40%) of the VSP detections **(Figure 3)**. The overwhelming majority of those disagreements were related to VSP noise artifacts, while the on-site telemetry was recording interpretable waveform data (n = 35; 90%) **(Figure 3)**. Accordingly, the telemetry captured normal sinus rhythm (13%), atrial arrhythmia (23%), ventricular arrhythmia (18%), and sinus bradycardia (36%) during VSP noise **(Table 2)**. Significantly, none of the above telemetry detections resulted in a CMU detection and notification. The CMU essentially learned to ignore baseline rhythm abnormalities with the help of feedback from the nursing staff. However, in 10% (four detections) of the discordance, the VSP recorded atrial arrhythmias that were not also detected by the CMU due to lead failure at the time of arrhythmia detection.

### CMU detections (traditional telemetry)

**Figure 4:**
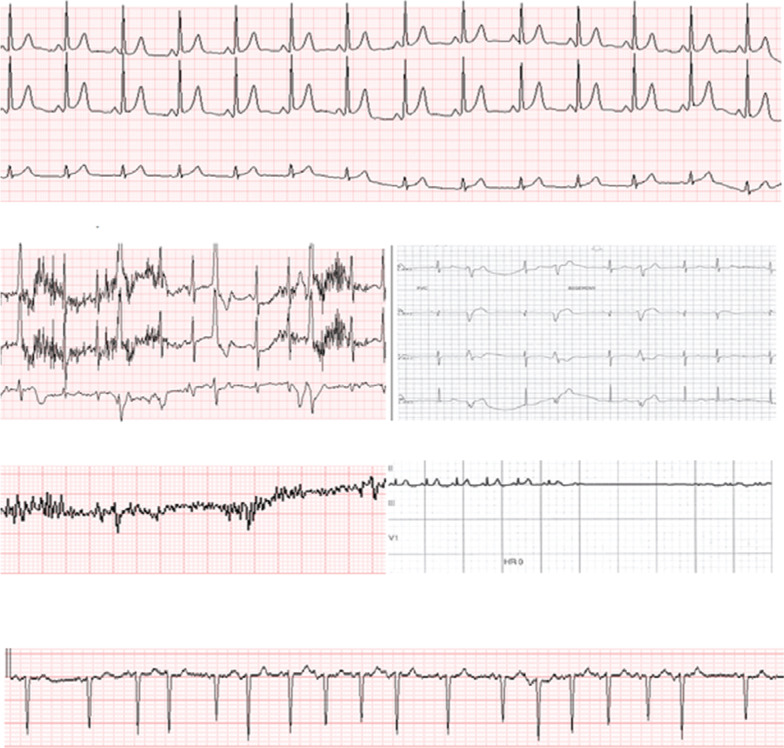
Sample of ECG waveform data featuring discordance between VSP and CMU detections. From top to bottom: (1) VSP normal sinus rhythm during CMU lead failure (no CMU data); (2) VSP noise during CMU ventricular arrhythmia; (3) VSP noise during CMU asystole; and (4) VAP atrial fibrillation undetected by CMU.

**Figure 5:**
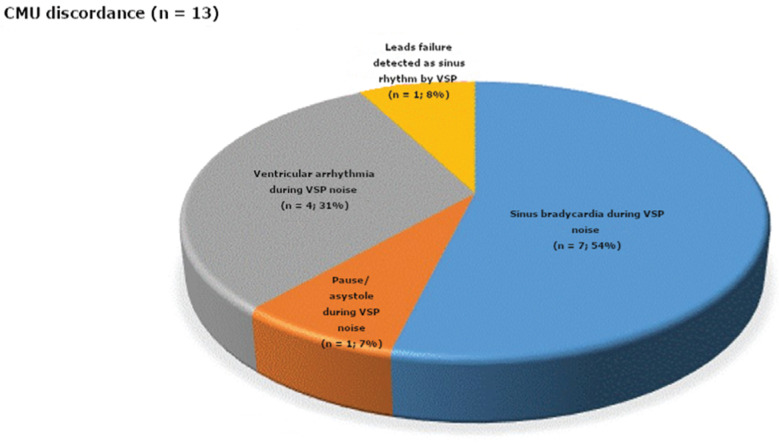
Pie graph characterizing the CMU detections that were discordant with VSP (n=13; 31%). Most resulted from VSP noise detected during sinus bradycardia (54%), pause/asystole (7%), and ventricular arrhythmia (31%). However, one case of discordance (8%) was due to an instance of CMU lead failure when the VSP detected sinus rhythm.

Out of the 42 CMU detections, there was discordance between the VSP and the CMU in the case of 13 (31%) **(Figure 4)**. Most of the instances of discordance (n = 12/13; 92%) were related to the availability of interpretable CMU waveform data during VSP noise **(Figure 5)**. The CMU detected ventricular arrhythmia (n = 4; 31%), sinus bradycardia (n = 7; 54%), and pause (n = 1; 7%) during VSP noise. The one remaining instance of CMU–VSP discordance (8%) was related with a VSP recording of sinus rhythm during a loss of signal in the CMU as a result of lead failure.

## Discussion

In this study, we sought to prospectively examine the feasibility and usability of the VSP CCRM system in non-ICU telemetry–monitored hospitalized patients, which is a group largely composed of patients with established heart rhythm disturbances who had just undergone an ablation procedure or who were electively admitted for monitored initiation of antiarrhythmic drug therapy to assess safety and efficacy. At a high level, the use of a novel patch-based monitor was successfully applied by nursing in all cases with relatively easy adaptation into their workflow and without any patch-related adverse events such as skin eruptions. Such is encouraging for the potential of patch-based hospital monitoring given widespread adoption in outpatient ambulatory ECG monitoring. However, the number of VSP detections (n = 98) was more than twice the number of CMU detections (n = 42), which is relevant in the era of heightened patient safety concerns regarding alarm fatigue.^3^ Furthermore, we determined that most of the discordance (90% of VSP discordance and 92% of CMU discordance) occurred because of VSP noise resulting in the loss of raw ECG data. Unfortunately, our study hypothesis was that the use of a patch-based skin fixation mechanism for ECG recording would improve the fidelity of waveform recording, not worsen it. However, the status quo of traditional telemetry monitoring again demonstrated a potential for improvement. In 10% of the VSP-triggered discordance, VSP-recorded atrial arrhythmias were missed by the CMU because of lead failure. Furthermore, in 8% of the CMU-triggered discordance, the VSP recorded normal sinus rhythm during lead failure. Thus, these preliminary findings suggest that a better patch monitor could potentially detect arrhythmia events missed by the status quo and also have recorded interpretable ECG data during the interruption of traditional telemetry detection.

To our knowledge, this is the first study to evaluate patch-based monitors in the inpatient setting. In a study by Barrett et al.^8^ comparing a 14-day monitoring protocol with the Zio patch (iRhythm Technologies, San Francisco, CA, USA) and conventional 24-hour Holter monitoring in the outpatient setting, the former detected 57% more clinically significant events but was found to be less sensitive in the detection of events during the first 24 hours of dual monitoring, when the multilead Holter connections were still very robust. In our study, the number of VSP detections was higher than the number of CMU detections because the VSP detected baseline rhythm abnormalities that were not generally clinically meaningful. Meanwhile, the CMU employed reprogrammed patient-specific alarm thresholds as determined by the nursing staff to mitigate the number of unactionable alarms, which is a routine care protocol in our institution. For example, one of the study patients experienced an elevated burden of premature ventricular contractions (PVCs) at baseline that did not require clinical action. Communication between the nursing and the CMU had resulted in a higher patient-specific threshold of 20 PVCs/minute or more for the alarm, which resulted in a marked alarm reduction for the CMU as compared with the VSP system for that patient, which used its nominal settings. It should be noted that this reflects the standard workflow of the CMU and could perhaps be judged to be unfair to the VSP given the locked-in nature of its nominal settings. A similar patient-specific alarm parameter detection method could be applied to future-state patch monitors in the instance of live patient monitoring. Thus, this barrier could be easily overcome.

### Study limitations

Our study has several limitations that are important to bring up. The first limitation was the feasibility sample size. This study could be completed only in 11 patients instead of the proposed 25 patients because the VSP device was withdrawn from the market by the manufacturer. The second limitation is that the study was conducted in a single center, which limits the generalizability of its findings.

## Conclusion

The principal aim of assessing the early feasibility and usability of patch-based monitoring for non-ICU patients receiving telemetry monitoring was achieved, but the overall results were quite disappointing. The authors continue to see opportunities to challenge the gold standard of conventional cardiac telemetry monitoring. However, it is abundantly clear that patch-based CCRM requires further development and refinement to replace traditional cardiac telemetry monitoring, with a focus on better fidelity of ECG recording. Still, the technology could one day evolve to detect clinically meaningful events missed by traditional methods if noise issues are mitigated. With the ongoing novel advances in both the areas of hardware and software in wireless cardiac monitoring technology, we believe that an opportunity to improve the existing telemetry infrastructure exists. An ideal patch monitor should have a high degree of recording fidelity, allow for real-time analysis of ECG waveforms, and be patient- and nursing-friendly. As patch monitoring–based technology continues to evolve, more large-scale trials are required to validate device safety, efficacy, and cost-effectiveness in relation to conventional monitoring.

## Figures and Tables

**Table 1:** Baseline Clinical Characteristics for the Study Cohort (n = 11)

Patient demographics	
Age	66.8 ± 12.5 years
Male gender	6 (55%)
Clinical characteristics	
Hypertension	5 (45%)
Diabetes	1 (9%)
Coronary artery disease	1 (9%)
History of AF/AFL	10 (90%)
Indication for telemetry	
Postelectrophysiology procedure	6 (55%)
Initiation of antiarrhythmic drug therapy	5 (45%)

AF: atrial fibrillation; AFL: atrial flutter.

Values are presented as either means ± standard deviations or n (%).

**Table 2:** Overview of all CMU- and VSP-triggered Events for the Study Patients (n = 11) Alongside the Results of Detection from the Corresponding Modality

Patient Number	CMU-triggered Event(s)	VSP Correlation?	VSP-triggered Event(s)	Telemetry Correlation (± CMU Notification*)?
1	None	N/A	2 noise events	2 cases of normal sinus rhythm
			4 noise events	4 cases of sinus bradycardia (no CMU notification)
2	None	N/A	None	N/A
3	None	N/A	None	N/A
4	2 sinus bradycardia events	2 cases of noise	10 noise events	10 cases of sinus bradycardia (no CMU notification)
5	1 asystole event	1 case of noise	1 noise events	1 case of normal sinus rhythm
6	3 sinus bradycardia events	3 cases of noise	3 atrial arrhythmia events	3 cases of lead failure (no CMU notification)
	1 ventricular arrhythmia event	1 case of noise	None	N/A
7	2 sinus bradycardia events	2 cases of noise	9 noise events	9 cases of atrial arrhythmia (no CMU notification)
8	None	N/A	1 noise event	1 case of normal sinus rhythm
9	None	N/A	None	N/A
10	1 ventricular arrhythmia event	1 case of noise	7 noise events	7 cases of ventricular ectopy (no CMU notification)
11	1 lead failure event	1 case of normal sinus	1 noise event	1 case of normal sinus rhythm
	2 ventricular arrhythmia event	2 cases of noise	1 atrial arrhythmia event	1 case of lead failure (no CMU notification)
Total	13	-	39	-

*In the case of VSP-triggered events, the last column also indicates whether the CMU provided clinical notification for what was detected on telemetry.
